# Novel Positional and Biological Candidate Gene for Grip Strength in Older Adults: The Long Life Family Study

**DOI:** 10.1093/geroni/igab046.533

**Published:** 2021-12-17

**Authors:** Adam Santanasto, Mary Wojczynski, Ryan Cvejkus, Lihua Wang, Bharat Thyagarajan, Kaare Christensen, E Warwick Daw, Joseph Zmuda

**Affiliations:** 1 University of Pittsburgh, Pittsburgh, Pittsburgh, Pennsylvania, United States; 2 Washington University School of Medicine, Washington University School of Medicine, Missouri, United States; 3 University of Pittsburgh, Pittsburgh, Pennsylvania, United States; 4 Washington University School of Medicine, St. Louis, Missouri, United States; 5 University of Minnesota, Minneapolis, Minnesota, United States; 6 Department of Public Health, University of Southern Denmark, Odense, Syddanmark, Denmark; 7 Washington University in St. Louis, St. Louis, Missouri, United States

## Abstract

Grip strength is a robust indicator of overall health, is moderately heritable and predicts longevity in older adults. Using genome-wide linkage analysis, we identified a novel locus on chromosome 18p linked to grip strength in 4534 individuals from 582 families (age 70.0 ± 15.8, range 24–110 years; 54% women). DNA sequencing was completed to identify single nucleotide variants (SNVs) in the 3.44 – 4.04 mega-basepair region on chromosome 18p. Using the sequencing data, we performed association analyses between the 7312 SNVs in the region and grip strength in families exhibiting evidence for linkage. Models were adjusted for age, age2, sex, height, field center and population substructure. There were 23 families (263 individuals) that contributed to the linkage peak (cumulative logarithm of the odds [LOD] score = 12.4). Six families (112 individuals) accounted for most of the linkage signal (LOD = 6.4). In these 6 families, we found highly significant associations between SNVs in the Disks Large-associated Protein 1 (DLGAP1) gene and grip strength (lead SNV: β= -0.75kg ± 0.15, p-value= 4.3*10-6). Correcting for the top SNV in DLGAP1 reduces the LOD by 72% in these families. Further, the effect allele frequency is much higher in these 6 families (39.7%) compared with both the NHLBI’s Trans-OMICs for Precision Medicine (23.5%) and 1000Genomes (28.0%) references panels. The DLGAP1 gene plays an important role in post-synaptic density of neurons; thus, it is a novel positional and biological candidate gene for follow-up studies aimed at uncovering genetic determinants of muscle strength.

